# Reliable Refuge: Two Sky Island Scorpion Species Select Larger, Thermally Stable Retreat Sites

**DOI:** 10.1371/journal.pone.0168105

**Published:** 2016-12-28

**Authors:** Jamie E. Becker, Christopher A. Brown

**Affiliations:** 1 Department of Biology, Bowling Green State University, Bowling Green, Ohio, United States of America; 2 Department of Biology, Tennessee Technological University, Cookeville, Tennessee, United States of America; University of Sao Paulo, BRAZIL

## Abstract

Sky island scorpions shelter under rocks and other surface debris, but, as with other scorpions, it is unclear whether these species select retreat sites randomly. Furthermore, little is known about the thermal preferences of scorpions, and no research has been done to identify whether reproductive condition might influence retreat site selection. The objectives were to (1) identify physical or thermal characteristics for retreat sites occupied by two sky island scorpions (*Vaejovis cashi* Graham 2007 and *V*. *electrum* Hughes 2011) and those not occupied; (2) determine whether retreat site selection differs between the two study species; and (3) identify whether thermal selection differs between species and between gravid and non-gravid females of the same species. Within each scorpion’s habitat, maximum dimensions of rocks along a transect line were measured and compared to occupied rocks to determine whether retreat site selection occurred randomly. Temperature loggers were placed under a subset of occupied and unoccupied rocks for 48 hours to compare the thermal characteristics of these rocks. Thermal gradient trials were conducted before parturition and after dispersal of young in order to identify whether gravidity influences thermal preference. *Vaejovis cashi* and *V*. *electrum* both selected larger retreat sites that had more stable thermal profiles. Neither species appeared to have thermal preferences influenced by reproductive condition. However, while thermal selection did not differ among non-gravid individuals, gravid *V*. *electrum* selected warmer temperatures than its gravid congener. Sky island scorpions appear to select large retreat sites to maintain thermal stability, although biotic factors (e.g., competition) could also be involved in this choice. Future studies should focus on identifying the various biotic or abiotic factors that could influence retreat site selection in scorpions, as well as determining whether reproductive condition affects thermal selection in other arachnids.

## Introduction

Many ectotherms behaviorally regulate body temperature within a fairly narrow range across microhabitats that differ substantially in temperature [1, 2]. Foremost among behavioral adaptations are the exploitation of hiding during the day and moving from sun to shade or vice versa, which either provides an escape from extreme temperatures [3] or a way to cool or heat the body. Maintaining a preferred body temperature improves an animal’s ability to carry out such activities as predator avoidance [4], prey capture [5, 6, 7, 8, 9], mate acquisition or defense [10, 11], resource ingestion and assimilation [12, 13, 14, 15, 16], and maintenance of optimum growth rate [11, 17, 18, 19], all of which affect the animal’s fitness. However, the preferred temperature may vary depending on the physiological state of the animal. For instance, temperature selection for digestion may differ from temperature selection for escape behaviors [4, 20].

Reproductive condition influences thermal preferences of many female ectotherms. Several studies have indicated that gravid females prefer higher body temperatures than non-gravid females or males [16, 21, 22, 23, 24] while others have observed the opposite relationship [25, 26]. Explanations for these observations relate to the female’s fitness. Lower body temperatures decrease metabolism and increase incubation time which enhances offspring size and, subsequently, survivability [26, 27]. Higher body temperatures shorten the gestation period by raising the metabolic rate [24], which could increase fecundity by reducing the time frame between reproductive events [26] or possibly decrease the locomotor costs associated with carrying larger offspring [28, 29]. While there is abundant literature regarding the relationship between thermal preferences and reproduction in reptiles, less is known for other groups of ectotherms, such as arachnids.

Scorpions are relatively secretive and inactive animals with a conservative repertoire of behaviors related to their feeding, shelter, and reproductive needs [30]. Scorpions are almost entirely nocturnal, typically spending most of their time in refugia that provide shelter from predation and extreme temperatures [4, 5]. Their high tolerance of extreme temperatures [3, 31, 32], low water loss rates [3, 33], and low metabolic rates relative to their body size [13] make them unique among ectotherms. However, few studies have investigated thermal preference in scorpion populations [2, 9, 29, 34, 35, 36].

Arizona and New Mexico contain a number of disjunct mountain ranges known as sky islands that make up a biogeographical corridor called the Madrean archipelago [37]. Each mountain range contains evergreen-oak woodlands at higher elevations and is surrounded by semi-desert grasslands at its base [38]. Many of these mountain ranges harbor endemic populations of small (approximately 20–30 mm), brown-colored scorpions in the genus *Vaejovis* that are found within the forested regions above 2000 m. These scorpions do not burrow, but instead dwell underneath rocks or pieces of bark during the day [39, 40], and emerge shortly after sunset to forage when ambient temperatures cool. Currently, it is not known whether *Vaejovis* retreat site selection is random, nor is there information about the mechanisms inherent in their selection, if any.

After a gestation period that lasts 6–9 months, sky island scorpions produce highly variable clutch sizes ranging from 5 to 45 scorplings per clutch, and average offspring mass ranges from approximately 1.85 to 2.45 mg [41]. Metabolic demands on the mother can thus fluctuate greatly, and females may wish to select predictable microclimates to better regulate metabolism. Different age groups of the same species (e.g., juveniles *vs*. adults) of scorpion appear to be maximally active at different temperatures [42], possibly implying that reproductive status may affect thermal preferences. But to our knowledge, only two publications part of the same study have aimed to understand whether gravidity affects temperature selection in Arizona bark scorpions [29, 36], and it is unknown whether reproductive condition might influence retreat site selection in other scorpions.

The objectives of this study were to identify factors influencing retreat site selection in the sky island scorpions *Vaejovis cashi* and *V*. *electrum*. The following questions are addressed: (1) Are there physical or thermal differences between cover objects occupied by scorpions and those that are not occupied?; (2) Does retreat site selection differ between species, either in physical characteristics of the retreat or in thermal characteristics?; and (3) Does temperature selection differ between gravid and non-gravid females of the same species?

## Materials and Methods

### Field Experiments

Because we wished to compare retreat site selection between two scorpion species, our study sites were located on rocky hillsides in the Chiricahua (31°54'33″ N, 109°14'56″ W, altitude = 1930 m) and Pinaleño (32°37' N, 109°48' W, altitude = 2502 m) Mountains, where endemic populations of *Vaejovis cashi* and *V*. *electrum* can be found, respectively. Sampling occurred in daylight from 3–8 June 2012, when adult females are in the final weeks before parturition. During this time of year, ambient temperatures in these areas can range from about 15–43°C [43], but soil surface temperatures can be much more extreme. For instance, at both of our sites, we observed maximum temperatures of 46°C under some retreat sites, and it is not uncommon for other semi-arid areas to have soil surface temperatures exceeding 45°C [3, 44].

To estimate abundance of potentially available retreat sites of various sizes within each study area, two 15 m long transects were laid down perpendicular to each other from an arbitrary focal point within each study area. Maximum dimensions (length and width, in cm) of all non-embedded rocks—these scorpions are unable to crawl under embedded rocks—touching a 1 m wide strip centered on the transect line were measured [17, 44]; transect rock measurements are hereafter referred to as “available” rocks. We did not turn rocks or collect scorpions within a transect, as this might introduce bias. We then searched the hillside intensively by turning rocks of all sizes. There were rare circumstances when a scorpion escaped collection (less than ten out of 103), but these occupied rocks are excluded from all analyses. In addition, once a scorpion was located [*V*. *cashi* (N = 48), *V*. *electrum* (N = 45)], it was placed in an individually marked vial, and the maximum dimensions (length, width, and depth, in cm) of the occupied retreat site was recorded.

Following collection of scorpions from each site, we measured thermal profiles under select rocks that either harbored scorpions (occupied rocks) or did not harbor scorpions (unoccupied rocks), using iButton temperature loggers (model DC1921G, Maxim Integrated, San Jose, USA). Because we had a limited number of iButtons (N = 30), we used the following procedure to select rocks so that we sampled across the entire size range for both occupied and unoccupied rocks. For occupied rocks, we first ranked these rocks by area (length × width) and then divided them into three groups with equal sample sizes. From each of these three groups, we haphazardly selected five rocks that were spatially separated in such a way that the majority of the area searched for scorpions was included. To select unoccupied rocks, we ranked the rocks from the two transects by area and divided them into three groups of equal sample size; we then used the range in area for each of these three groups as our basis for selecting rocks. We haphazardly selected five locations spread across our sampling area, and within each of these locations we randomly chose one rock from each of the three size range categories obtained from the transect rock data. Within a location, each selected rock was a minimum of 1 m from another selected rock.

After rocks to be sampled were chosen, we measured length, width, and depth of each occupied and unoccupied rock. We then placed an iButton under the approximate center of each rock, buried so the top of the iButton was flush with the surface. All rocks were flagged with an identifying label. Temperatures were recorded every 30 min for 48 hours, after which the iButtons were retrieved.

### Laboratory Procedures

Female *V*. *cashi* and *V*. *electrum* give birth from July—October, and because we sampled in early June, we were able to easily determine which individuals were gravid. Gravid females appear engorged to the point where there are large spaces between their sclerites (S1 Fig), and their large litter size (15–25 scorplings, on average) can not only be seen through the body wall, but it causes a substantial weight gain (*V*. *cashi*: 23–31 mg; *V*. *electrum*: 44–62.5 mg; [41]). Using Steffenson and Brown’s [41] data, we presumed that *V*. *cashi* and *V*. *electrum* could be considered gravid if they weighed more than 50 or 130 mg, respectively. Once scorpions were weighed at SWRS immediately after capture, we discovered significant differences between individuals that proved to be gravid (*V*. *cashi*: N = 40; *V*. *electrum*: N = 32) and non-gravid (*V*. *cashi*: N = 5; *V*. *electrum*: N = 12) both within (P < 0.001) and between (P < 0.001) species (S2 Fig). We then transported scorpions to Tennessee Technological University (TTU) in Cookeville, TN, and housed them individually in square Petri dishes (9 x 9 x 1.5 cm) that had a sand substrate with a square paper towel (approximately 4 cm^2^) for cover. Petri dishes were placed into large plastic bins with moistened paper towels covering the bottom; this method provided some humidity without directly introducing moisture into the scorpion’s enclosure. Containers were cleaned if soiled, and scorpions were fed one juvenile cricket (2^nd^-3^rd^ instar) each week. Scorpions were maintained at 20°C ± 3°C with access to natural lighting and were acclimated six weeks before experiments began. We assumed that a gravid female would wish to spend the longest amount of time at a preferred temperature to maximize reproductive effort. Retreat sites of different sizes can offer similar maximum and minimum temperatures, but they will differ in the length of time mid-range temperatures are offered (i.e., variance in temperature; see results). Because of this crucial difference, and because we did not have the capability to slightly reduce temperatures below 20°C ± 3°C, we selected a gradient that best represented the mid-range temperatures observed in the field (S6 and S7 Figs; see discussion). Following Webber and Bryson’s [45] design, we constructed a chamber (S3 Fig) that ranged from 23°—46°C. The dimensions of each chamber measured 76 x 18 x 18 cm, and a sandy substrate was used in lieu of a gravel substrate to have more gradual temperature increases and to more closely resemble environmental conditions. Seven ceramic tiles (10.2 x 10.2 x 0.95 cm) were evenly placed along the length of each chamber to serve as cover objects. Each chamber varied slightly in its thermal range because of the spatial distribution of sand, so eight iButtons were placed along the length of each chamber—see S3 Fig for iButton positions—to accurately measure temperature.

Laboratory trials were performed from 10 July to 17 September 2012. Non-gravid individuals (N = 26, including males), and gravid females (N = 36) were placed individually in chambers at the cooler end and given a 24-hour acclimation period. The scorpion’s position was recorded three times a day—early morning (0900–1000 h), late afternoon (1500–1700 h), and late evening (2100–2200 h)–for one to three days following acclimation. By correlating iButton data with a scorpion’s position, average temperature selection for each scorpion could be calculated. Following a trial, the sand was replaced and the chambers and tiles were wiped with alcohol to remove any chemical cues that could influence the next scorpion’s decision. After females had already completed trials in their gravid state, thermal gradient trials were repeated for the same females (N = 14) six to thirty days following offspring dispersal. Scorpions experienced high mortality during and between trials which resulted in shorter trial periods (e.g., one day *vs*. three days) and low sample sizes for the post-partum trials.

### Analyses

All data were analyzed using SPSS (version 19.0); normality and homogeneity of variance were tested using Kolmogorov-Smirnov and Levene’s tests, respectively. Statistical significance was set at α = 0.05 (two-tailed). To satisfy parametric assumptions, some data were log-transformed, and non-parametric tests were conducted when parametric assumptions were not met.

Surface area of transect rocks was compared between study areas using a *t*-test to compare habitat availability; surface area and depth of occupied and unoccupied rocks were compared between study areas using either a *t*-test or a Mann-Whitney U test. Because occupied and unoccupied rock size classes from each study area differed substantially, it was necessary to define a single set of size classes for further analyses. The new set of size classes [very small (< 500 cm^2^), small (500–1000 cm^2^), medium (1001–2000 cm^2^), and large (> 2000 cm^2^)] was defined so that approximately equal numbers of occupied rocks (7–8 per size class, combining both sites) occurred in each size range. Rocks in other categories (e.g., transect rocks, all occupied rocks, and unoccupied rocks with iButtons under them) were then assigned to these new size classes. To determine whether scorpions selected retreat sites randomly, a chi-square test compared the percentage of occupied retreat sites of various size classes (based on the new category) to what would be expected, given the available size distribution within a study area. Thermal characteristics—mean (μ), temperature change (ΔT), and variance (σ^2^)–of occupied and unoccupied rocks from each size class were compared using either a *t*-test or a Mann-Whitney *U* test to understand how the thermal environment changes according to retreat size.

Huey et al. [44], divided rocks into three thickness size categories (< 20 cm, 20–40 cm, and > 40 cm) for thermal analysis. However, the majority of rocks at the Chiricahua and Pinaleño sites fell into the ‘< 20 cm’ category, and we thought it appropriate to divide rock thicknesses into two categories that represented the available retreat sites. Therefore, for both occupied or unoccupied groupings, thermal characteristics were compared (using Mann-Whitney *U* tests) between only two rock thickness size classes (< 10 cm and > 10 cm).

Analyses from laboratory experiments employed either a *t*-test or Mann-Whitney *U* test when comparing thermal preferences between groups. A repeated measures *t*-test or a Wilcoxon signed-ranks test ascertained whether reproductive condition alters thermal preference in individuals.

### Ethics Statement

Scientific collection of invertebrate animals does not require an official permit; however, the Southwestern Research Station and the Arizona Game and Fish Department provided permission to collect scorpions at our two study locations. No endangered or protected species were at risk during our study.

## Results

Rocks at the Chiricahua site were smaller than those at the Pinaleño site in both surface area (Table 1; *t*_971_ = 8.93, *P* < 0.001) and depth (*U* = 52.0, N = 30, *P* = 0.012). Unoccupied rocks from the Pinaleño Mountains did not differ in surface area from transect rocks (*t*_152_ = 1.68, *P* = 0.096). However, unoccupied rocks from the Chiricahua Mountains were larger in surface area than transect rocks (*t*_848_ = 2.87, *P* = 0.004). Preferences of rock sizes were not equally distributed among the four size categories for *V*. *electrum* (*X*^*2*^ *=* 294, df = 3, N = 43, *P* < 0.001) or *V*. *cashi* (*X*^*2*^ *=* 599, df = 3, N = 48, *P* < 0.001). Very small rocks composed 84.2% of transect rocks at the Pinaleño site and 94.7% of transect rocks at the Chiricahua site. However, *V*. *electrum* and *V*. *cashi* selected against very small rocks (S4 Fig; Standardized residuals [SR] = -5.52 and -5.11, respectively), while the other three rock sizes were selected more often than expected (small: SR = 3.50 and 12.5; medium: SR = 13.9 and 19.1; large: SR = 7.48 and 8.58). *Vaejovis electrum* selected rocks with a larger surface area (*t*_89_ = 2.92, *P* = 0.004), and they selected slightly thicker (but not significantly so) rocks (*t*_89_ = 1.36, *P* = 0.18) than *V*. *cashi*.

**Table 1:** Physical Characteristics for Transect (T), Occupied (O), and Unoccupied (U) Rocks.

		Pinaleño Mtns.		Chiricahua Mtns.	
Surface area (cm^2^)		μ ± SE	n	μ ± SE	n
	**T**	345 ± 42.8	139	143 ± 9.36	834
	**O**	1464 ± 140	43	1015 ± 89.3	48
	**U**	597 ± 172	15	470 ± 180	15
**Depth (cm)**					
	**O**	15.3 ± 1.07	43	13.8 ± 1.17	48
	**U**	8.03 ± 1.30	15	4.40 ± 1.46	15

**Fig 1:**
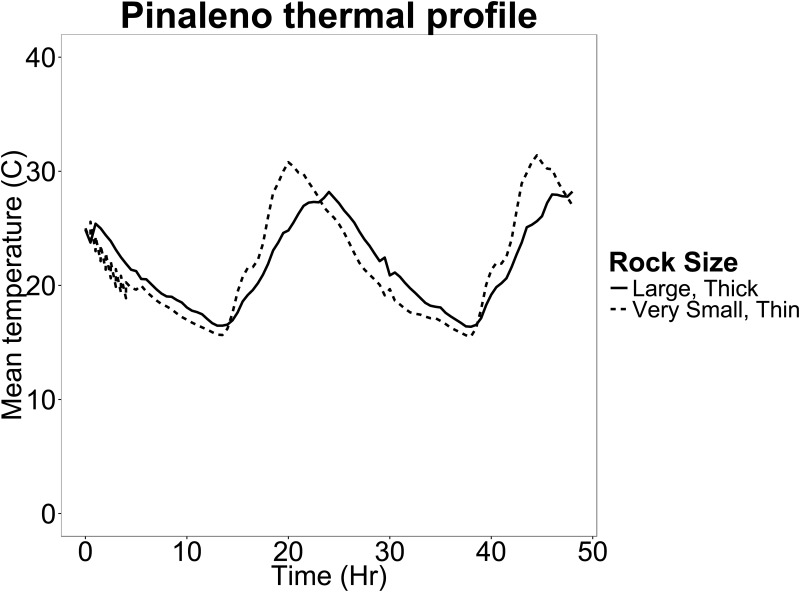
Thermal Profiles. This figure shows mean temperature of all very small, thin rocks (dotted line) and all large, thick rocks (solid line) from the Pinaleño site. Note that large, thick rocks provide more stable temperatures, while small, thin rocks more closely resemble daily ambient temperature fluctuations. See S6 and S7 Figs to view all 30 thermal profiles recorded from these two rock size classes.

Thermal characteristics among the four rock size classes differed substantially (Table 2, Figs 1 and S5–S7). Very small rocks had a significantly higher ΔT and σ^2^ than the remaining three rock sizes, and small rocks had a significantly higher ΔT and σ^2^ than medium rocks but did not differ from large rocks for these two variables. Furthermore, medium rocks did not differ significantly from large rocks for these two variables.

**Table 2:** Pairwise Comparisons of Thermal Characteristics.

Rock Size Class	Temperature Variable	Small	Medium	Large
**Very small**	μ	40.0	29.0*	31.0
	ΔT	24.0**	11.0***	15.0**
	σ^2^	31.0*	16.0***	17.0**
**Small**	μ		49.0	42.0
	ΔT		20.5**	37.5
	σ^2^		31.0*	38.0
**Medium**	μ			44.0
	ΔT			34.0
	σ^2^			47.0

Numbers in cells are *U*-values. A Kruskal-Wallis ANOVA indicated a significant difference for ΔT (H_3_ = 19.1, N = 44, *P* < 0.001) and variance (H_3_ = 14.4, N = 44, *P* = 0.002) among the four rock size classes, but μ did not significantly differ among the four rock size classes (H_3_ = 7.0, N = 44, *P* = 0.071).

*P < 0.05;

**P < 0.01;

***P < 0.001.

ΔT (*U* = 67.5, N = 40, *P* < 0.001) and σ^2^ (*U* = 88.0, N = 40, *P* = 0.002), but not μ (*U* = 140, N = 40, *P* = 0.11), were significantly higher in thin than thick rocks (S5 Fig). Thermal characteristics of thin rocks were similar to very small rocks (μ: *U* = 88.5, N = 32, *P* = 0.22; ΔT: *U* = 77.5, N = 32, *P* = 0.098; σ^2^: *U* = 77.5, N = 32, *P* = 0.098).

Within the Pinaleño study area, μ (*t*_28_ = 1.47, *P* = 0.15), ΔT (*t*_28_ = 2.95, *P* = 0.006) and σ^2^ (*t*_28_ = 2.76, *P* = 0.010) were greater in unoccupied than occupied rocks. Within the Chiricahua study area, μ (*U* = 109, N = 30, *P* = 0.89), ΔT (*U* = 73.0, N = 30, *P* = 0.10) and σ^2^ (*U* = 85.0, N = 30, *P* = 0.25) did not differ between occupied and unoccupied rocks.

**Fig 2:**
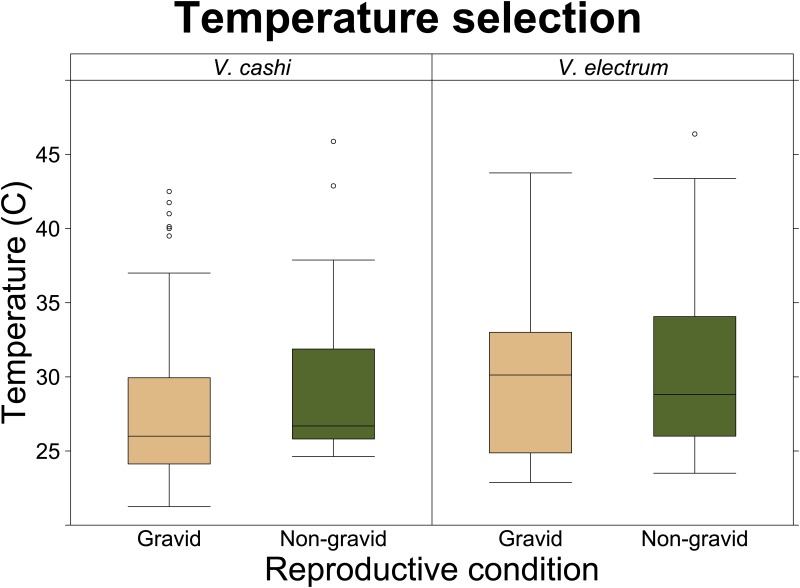
Temperature Selection. This figure shows temperature selection of all gravid and non-gravid individuals of each species, including those that have already experienced trials as both gravid and non-gravid states. Extreme variability is likely due to a combination of individual differences and exploratory behavior.

As a whole, gravid *V*. *electrum* (*n* = 19, mean ± SE = 29.7 ± 0.68°C) chose higher temperatures (*U* = 85.0, N = 36, *P* = 0.015) in laboratory conditions than gravid *V*. *cashi* (*n* = 17, 27.3 ± 0.39°C) (Fig 2), and thermal selection of non-gravid *V*. *electrum* (*n* = 15, 29.8 ± 0.53°C) did not differ (*t*_24_ = 0.98, *P* = 0.34) from non-gravid *V*. *cashi* (*n* = 11, 29.0 ± 0.62°C). In a different comparison, *V*. *electrum* did not alter their thermal preference after parturition (*n =* 7, gravid: 30.6 ± 1.30°C, non-gravid: 29.5 ± 0.68°C, *t*_6_ = 0.86, *P* = 0.42), nor did *V*. *cashi* (*n =* 7, gravid: 27.7 ± 0.58°C, non-gravid: 29.0 ± 0.92°C, *t*_6_ = 1.82, *P* = 0.12). Please see S1 File for all data presented here.

## Discussion

### Physical and Thermal Characteristics of Retreat Sites

In general, *V*. *electrum* and *V*. *cashi* selected a greater proportion of larger sized rocks than what would be expected based on what was available in their habitat. *Vaejovis electrum* selected rocks with a significantly larger surface area than *V*. *cashi*, but there was no significant difference between rock thicknesses occupied by these two species.

Biotic interactions (with conspecifics, predators, prey, etc.) can be directly influenced by rock size [17]. The low level of surface activity observed in scorpions may ultimately be attributed to strong predation pressure [46], since many vertebrates and invertebrates prey on scorpions [47]. It follows that large rocks may provide better cover from some predators; for example, the weight of larger rocks may restrict the ability of predators (e.g., procyonids) to overturn them. Intraspecific competition for shelter may also affect the spatial distribution of scorpions [48]. *Vaejovis electrum*, being a larger scorpion than its Chiricahuan congener, might require larger-sized rocks to reduce competition, although competitive interactions among *Vaejovis* scorpions are largely unknown [13].

However, rock size also influences abiotic properties under them. Thermal characteristics of cover objects are also probably influential in the selection of appropriate retreat sites of sky island scorpions. Goldsbrough et al. [17] indicated that thermal stability offered by greater rock thickness and shelter area were important factors in retreat site selection in flat rock spiders, *Hemicloea major*. Others have shown that temperatures under rocks depend upon rock thickness [7, 8, 44]. Since *V*. *electrum* selected larger rocks than *V*. *cashi*, *V*. *electrum* consequently selected rocks that were significantly cooler and less variable in temperature. Although Pinaleño occupied rocks were approximately 2.8°C cooler than Chiricahua occupied rocks, *V*. *electrum* does not necessarily prefer cooler, less variable temperatures than *V*. *cashi*. The Chiricahua hillside had a larger percentage of very small rocks present; it is possible that *V*. *cashi* simply selected retreat sites to lessen search time. As search time increases, not only is the animal exposed to predators, but it is also at risk of suffering from heat stress, since soil surface temperatures can be double the air temperature in semi-arid habitats [49]. It should be noted that different mineral compositions could potentially alter thermal properties in rocks of equivalent size, but all rocks in our study areas were of similar composition.

Rocks of all size classes had similar mean temperatures, but ΔT and σ^2^ significantly differed from each other. In general, very small and thin rocks had similar ΔT and σ^2^, which were both much greater than the remaining rock size classes. Of the rocks for which soil moisture was measured, all had about 0% to 5% (± 5%) moisture content; therefore, soil moisture is not likely to be a variable that influences thermal characteristics of rocks, or habitat choice by scorpions, at these two sites. Since most studies only examined rock thickness rather than surface area [7, 8, 44], and only one known study suggests that rock thickness primarily determines thermal profiles underneath rocks ([46], as cited in [7]), it is unclear to what extent surface area or thickness alone contribute to soil surface temperatures underneath rocks. The results of this study suggest, however, that thermal regimes of a scorpion’s microhabitat are likely influenced both by rock surface area and thickness, as well as other potential variables such as percent canopy cover.

Since sky island scorpions are nocturnal, and do not burrow but instead hide under rocks during the day, they are not able to thermoregulate as efficiently as other ectotherms because they are limited to a small thermal range [44]. For instance, burrowing or crevice-dwelling species are able to enjoy the benefits of a much larger thermal range within a single retreat site [3, 7, 49] and can simply move throughout their retreat to achieve optimal metabolic rates for any physiological need. Selecting retreat sites that are too small (e.g., very small or thin rocks) could subject scorpions to extreme hot and cold temperatures which could severely impair their physiological processes [44]. Scorpions may therefore be more likely to select larger retreat sites with moderate thermal ranges in order to more easily regulate their metabolic processes within the highly stressful desert environment.

Maintenance of body temperature near a preferred optimum should enhance an animal’s fitness [19, 22]. Hotter temperatures tend to increase the metabolism of ectotherms, which accelerates digestion, water loss rates, growth and development [17, 27], gestation period [22, 24], and defensive behaviors such as sprint or sting speed [4]. However, an animal’s needs are constantly in flux, and optimum temperatures for a single trait may differ greatly from optimum temperatures from another trait. For example, avoidance of predators appeared to be a higher priority than thermal regulation in a rock dwelling gecko, *Oedura lesueurii* [50]. When food resources are scarce, selecting warmer temperatures for accelerated growth and reproduction may not be beneficial. Rather, the animal would increase fitness by selecting cooler temperatures to slow metabolism, and several studies have shown that hunger appears to interfere with thermoregulation [12, 14, 15, 51]. Similarly, when food is plentiful (e.g., in captivity), an ectotherm might enhance fitness by selecting hotter temperatures.

A scorpion may also indirectly benefit from selecting rocks with stable thermal regimes, which may be important in influencing the behavior of an animal’s prey [52]. For instance, antlions seem to be active at almost the same temperature range of their prey [53], even selecting retreat sites with temperatures appearing to be hotter than their preferred range [6]. The burrowing scorpion, *Opisthophthalmus latimanus*, modifies its behavior by stilting so that it may tolerate near-lethal temperatures during the day to catch prey that wander near the entrance [54]. Some potential prey items of scorpions (e.g., invertebrates that lack a thick waxy cuticle) are less resilient to extreme temperatures [3, 55], so occupying retreat sites that are likely to attract potential prey items (e.g., large rocks with stable temperatures) is important for a sit-and-wait predator. A scorpion may enhance its overall fitness by selecting seemingly suboptimal thermal ranges in order to attract prey.

### Thermal Selection

Since gravid *V*. *electrum* selected hotter temperatures than gravid *V*. *cashi*, it might be expected that non-gravid *V*. *electrum* would also select hotter temperatures than non-gravid *V*. *cashi*, but they, in fact, did not differ significantly from each other. *V*. *electrum* produces a larger litter size with a greater offspring mass than *V*. *cashi* [41]. While non-gravid congeners may have similar metabolic needs, larger species may have greater reproductive demands, resulting in selection of higher temperatures to maximize reproductive output. On the other hand, paired tests comparing individuals before and after parturition indicated that reproductive condition had no impact on thermal preferences in either species. Scorpions may need to accurately maintain the correct metabolic rate for other physiological processes and cannot afford to select different thermal regimes at each reproductive state.

It should be noted that scorpions may be more inclined to select warmer temperatures in the laboratory as a result of increased metabolic demands associated with ample food, as opposed to limited food resources likely present in field conditions. In addition, scorpions had a high mortality in captivity, and compromised health could be caused by stress or other factors which could influence efficiency in detecting or adapting to thermal changes in the environment. Additionally, some scorpions explored the thermal gradient, but others remained in one position throughout the entire trial period; thermal selection in the gradient may consequently be partially dependent upon escape behavior or random chance of a scorpion’s position at observation. Thus, reproductive status could very well be important for thermal selection [9, 29], but a large number of unavoidable factors contributed to an inability to detect real differences.

However, one potential issue with our design is that the low temperature produced by the thermal gradient was near the mean temperature found under occupied and unoccupied rocks, which could indicate that scorpions were unable to accurately select their preferred temperatures. We later validated our gradient in June 2016 by measuring body temperature of a scorpion’s dorsal surface (N = 36) with an IR thermometer (Southwire Tools Model 30010S, Carrollton, GA) immediately after turning a roadside or hillside rock near our site in the Chiricahua Mountains. We made sure to take measurements during the same time of day as had been done in our original study. We found that while the mean rock temperatures are at the low end of our gradient, scorpion body temperature is higher than this (roadside: 26.61 ± 0.81°C, hillside: 25.71 ± 1.05°C, unpublished data). Since a primary characteristic of rock size is its thermal variability (i.e., the time spent at a particular temperature), regardless of the mean, it is likely that a scorpion would choose to spend the majority of its time avoiding extremely high or low temperatures that are found under smaller sized rocks.

Our results clearly indicate a preference for larger rock sizes, although there are numerous potential biotic and abiotic explanations that likely interact with each other in complex ways [56]. Future studies should continue to examine thermal preference in association with reproductive demand, as well as the interaction with other physiological states. Other studies should attempt to discover some of the biotic relationships associated with physical and thermal characteristics of retreat sites. These questions not only have academic value, but could help us understand similar relationships in other arachnids, a vital intermediate predator in many food webs.

## Supporting Information

S1 Fig Gravid scorpion. Scorpions carry 15–25 offspring, on average, per litter (Steffenson and Brown 2013). Her body swells to accommodate her large litter size to the point where her cuticle is easily seen between her sclerites.
(JPG)

S2 Fig Determining Reproductive Condition via Body Mass. Scorpion body mass was weighed at the Southwestern Research Station immediately after collection to determine gravidity. Scorpions produce a large litter size, and each offspring can weigh anywhere from 1.85 to 2.45 mg [42]. As a result, females can double their body mass when gravid.
(TIFF)

S3 Fig Thermal Gradient Chamber. Thermal gradients that display the positions of iButtons, pebbles, and tiles. The cool end of the chamber is located at the top of the photo, while the hottest end of the chamber is located at the bottom. We allowed gaps between each tile, wall, and substrate to limit uneven air temperature that could interfere with thermal selection. Seven iButtons were located underneath the center of each tile. An eighth iButton, which had a similar thermal range as position 3, was placed in the corner at the hot end because scorpions often remained there for the duration of a trial.
(TIF)

S4 Fig Standardized Residuals of Retreat Surface Area. Scorpions selected rocks of small, medium, and large size more than what would be expected, given the available size range of rocks per study area.
(TIF)

S5 Fig Correlations Between Rock Size and Thermal Stability. Chiricahua rocks are represented as open circles, while Pinaleño rocks are represented as closed circles. Note how thermal σ^2^ and ΔT decrease as rock size increases, while mean temperature remains relatively the same. However, R^2^ values are low (see Fig 2 for explanation): (A) Pinaleño R^2^ < 0.001, Chiricahua R^2^ = 0.011; (B) Pinaleño R^2^ = 0.179, Chiricahua R^2^ = 0.094; (C) Pinaleño R^2^ = 0.287, Chiricahua R^2^ = 0.097.
(TIF)

S6 Fig Thermal profiles of large, thick rocks. This graph represents temperature readings over a period of 48 hours. Although all sites were subject to the same rock size classification, site separation was necessary because of differences in elevation and canopy cover. Because the Pinaleño site had larger, thicker rocks than the Chiricahua site, more data was available, and the thermal profiles are substantially less variable than the Chiricahua thermal profiles.
(TIFF)

S7 Fig Thermal profiles of very small, thin rocks. Like S6 Fig, this graph represents temperature readings over a period of 48 hours, and sites were separated because of differences in elevation and canopy cover. Because the Chiricahua site had a larger percentage of very small rocks, this site has more data available. However, note that very small, thin rock thermal profiles have significantly more variability than large, thick rocks.
(TIFF)

S1 File Supplemental data. This file contains all data presented in this paper. Specifically, this file includes 1) the dimensions and size classifications of each transect rock, occupied, unoccupied, and rock under which an ibutton was placed, 2) the scorpion’s weight at time of capture that corresponds to each occupied rock, 3) the temperature of each rock in the field at 30-minute intervals, 4) all lab data including individual thermal preferences at each time interval, 5) 2012 Tuscon weather data obtained from the National Weather Service [44], and 6) body temperature of *V*. *cashi* scorpions on the roadside and hillside in the Chiricahua mountains.
(XLSX)
