# Supplementary figures and images for: Reliable Refuge: Two Sky Island Scorpion Species Select Larger, Thermally Stable Retreat Sites
Source: PLoS One. 2016 Dec 28;11(12):e0168105. doi: 10.1371/journal.pone.0168105 (PMC5193400; doi:10.1371/journal.pone.0168105)

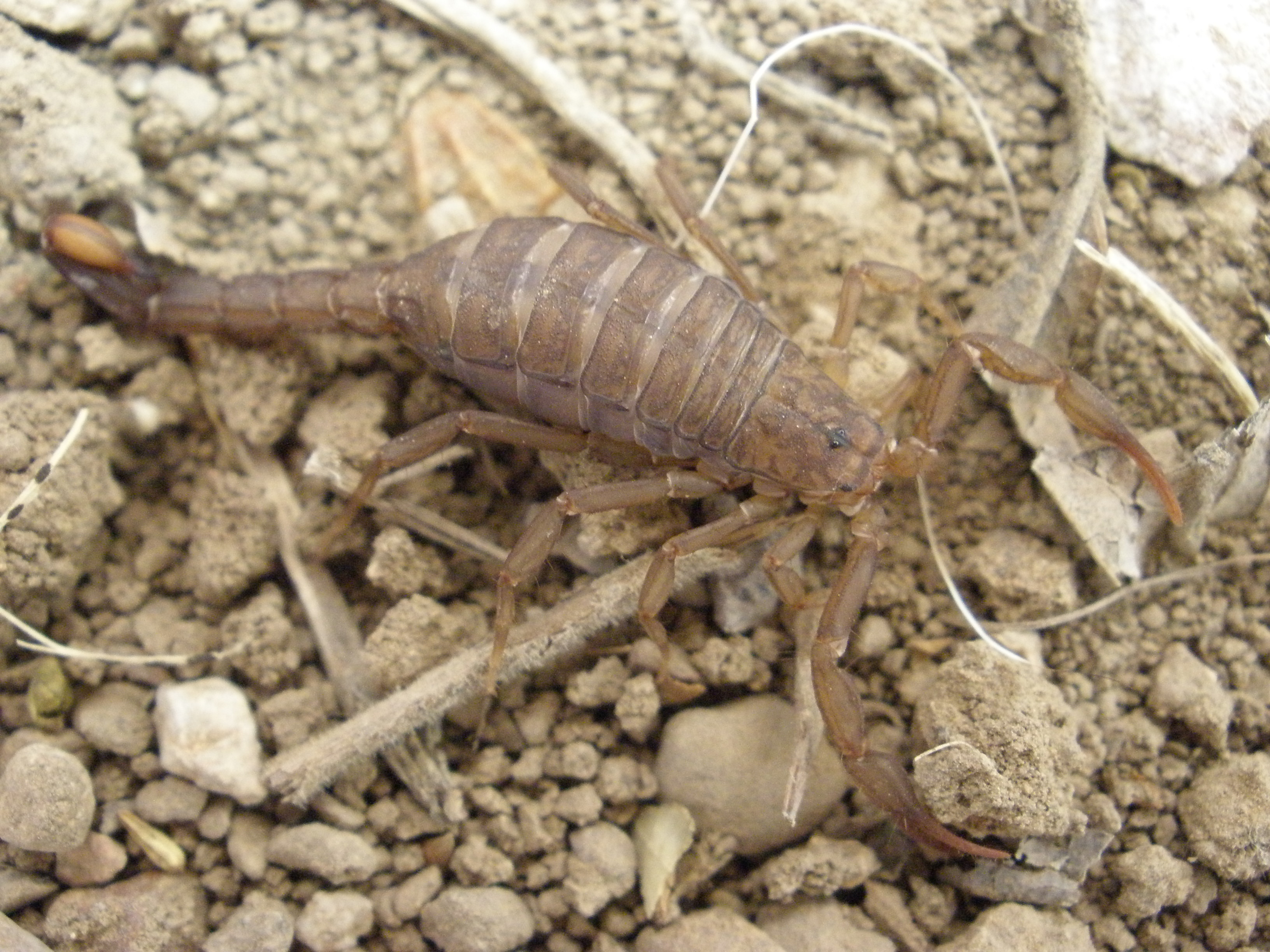

Supplement: S1 Fig — Scorpions carry 15–25 offspring, on average, per litter (Steffenson and Brown 2013). Her body swells to accommodate her large litter size to the point where her cuticle is easily seen between her sclerites. (JPG) [file pone.0168105.s001.JPG]

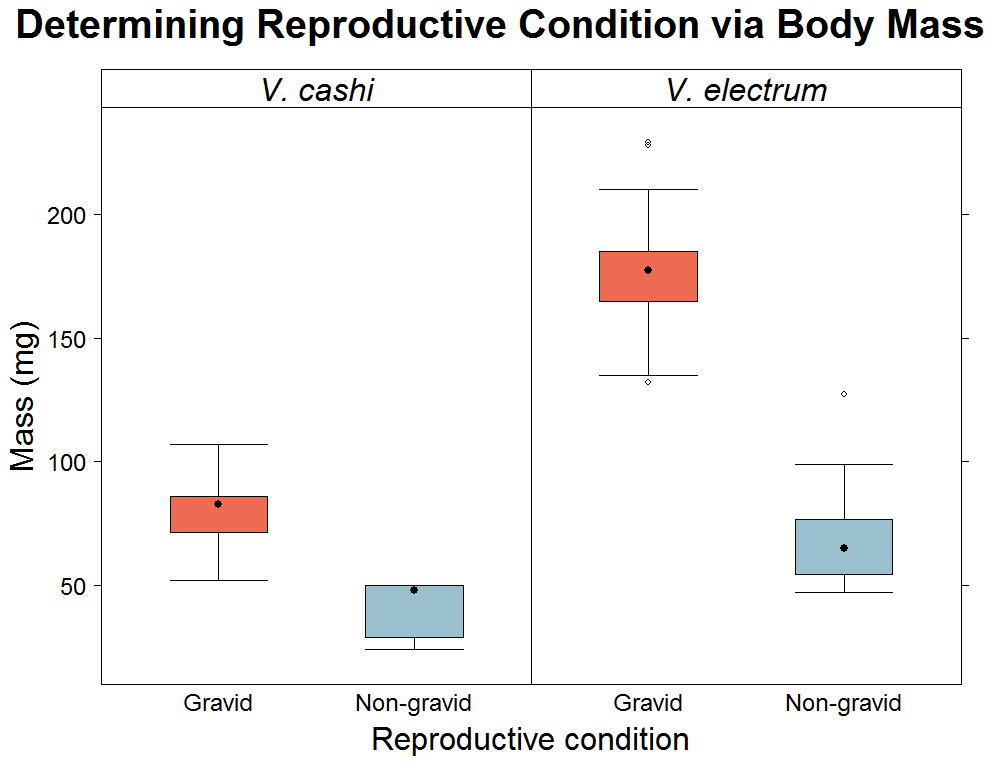

Supplement: S2 Fig — Scorpion body mass was weighed at the Southwestern Research Station immediately after collection to determine gravidity. Scorpions produce a large litter size, and each offspring can weigh anywhere from 1.85 to 2.45 mg [42]. As a result, females can double their body mass when gravid. (TIFF) [file pone.0168105.s002.tiff]

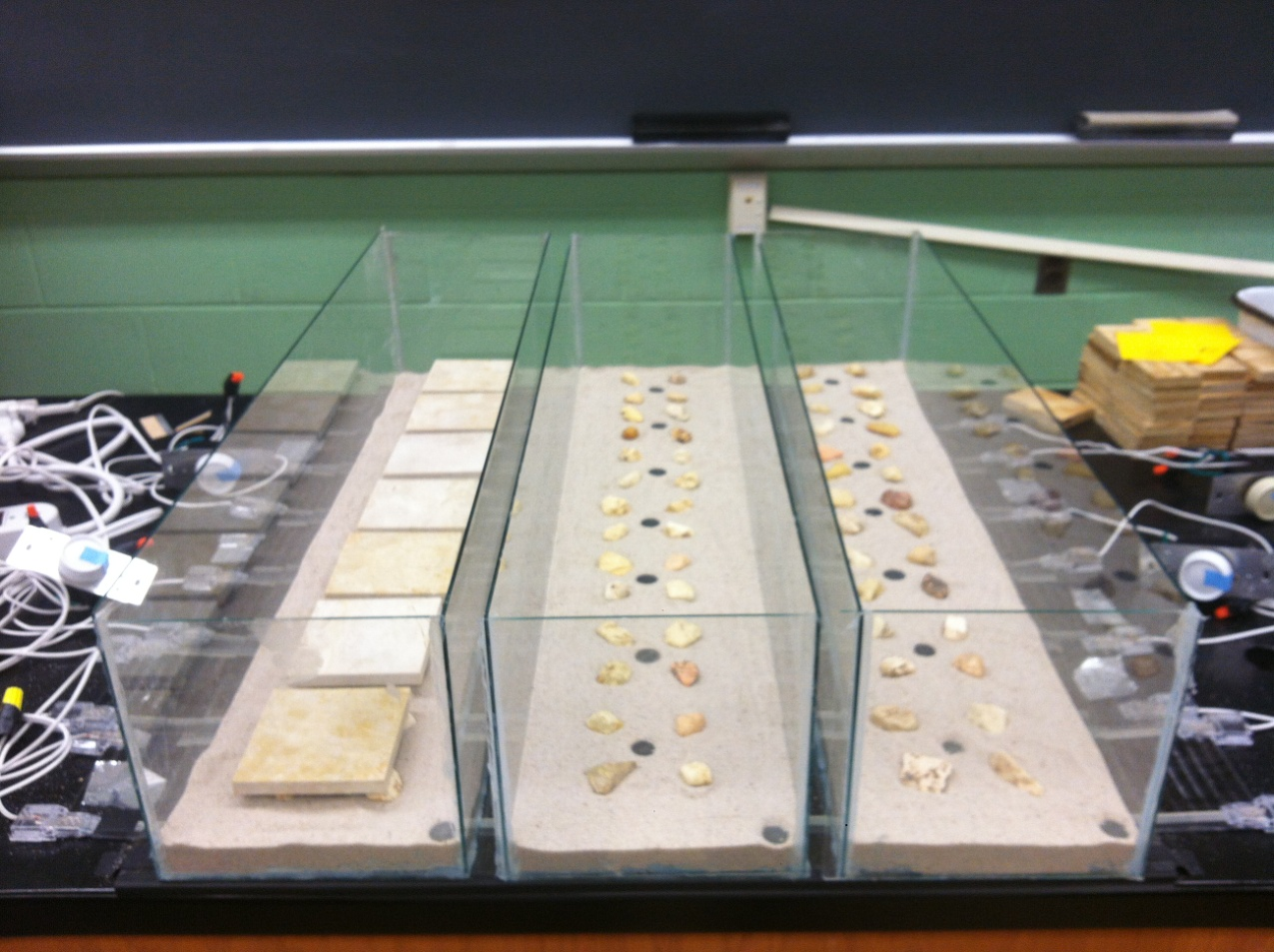

Supplement: S3 Fig — Thermal gradients that display the positions of iButtons, pebbles, and tiles. The cool end of the chamber is located at the top of the photo, while the hottest end of the chamber is located at the bottom. We allowed gaps between each tile, wall, and substrate to limit uneven air temperature that could interfere with thermal selection. Seven iButtons were located underneath the center of each tile. An eighth iButton, which had a similar thermal range as position 3, was placed in the corner at the hot end because scorpions often remained there for the duration of a trial. (TIF) [file pone.0168105.s003.tif]

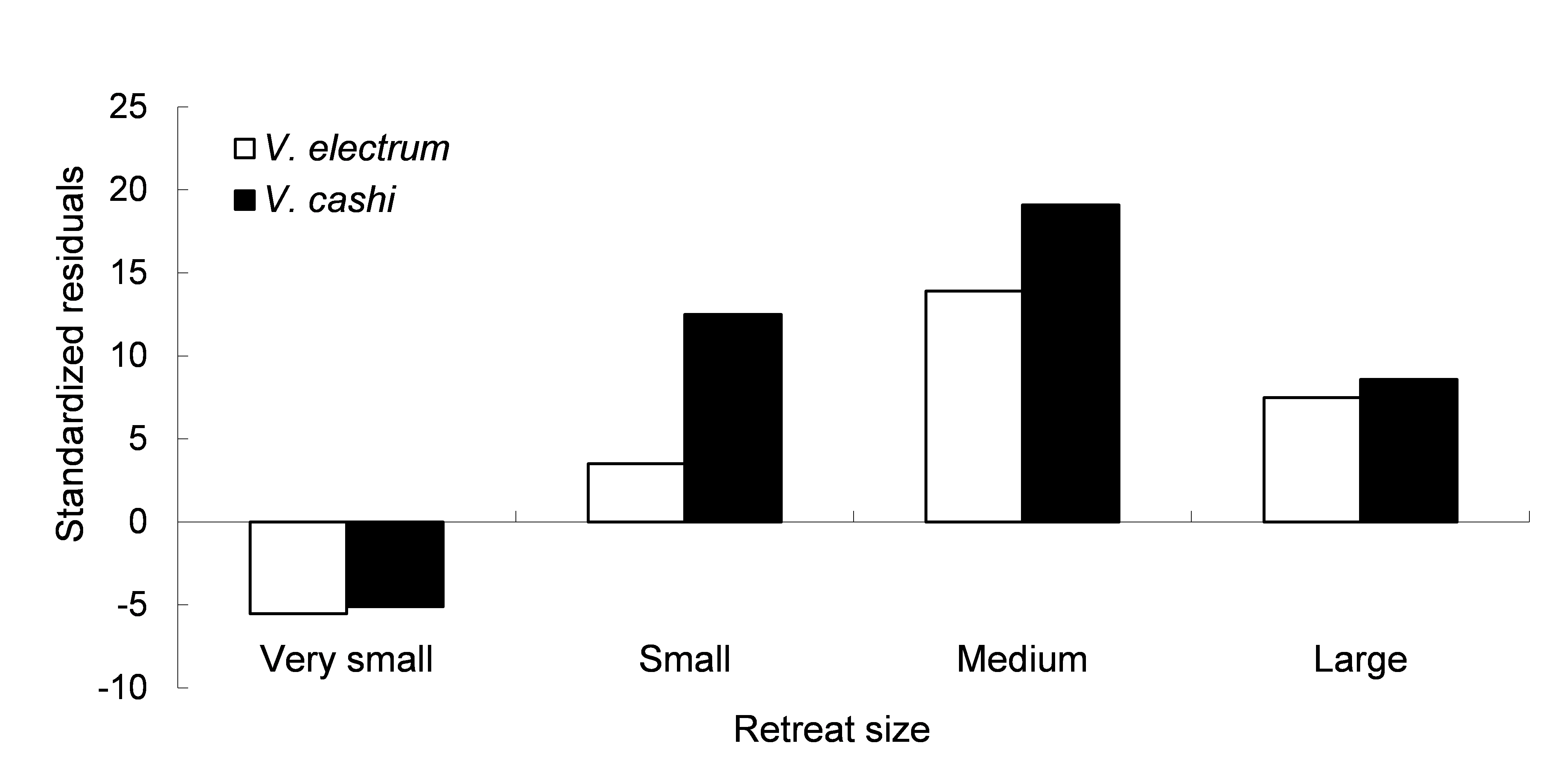

Supplement: S4 Fig — Scorpions selected rocks of small, medium, and large size more than what would be expected, given the available size range of rocks per study area. (TIF) [file pone.0168105.s004.tif]

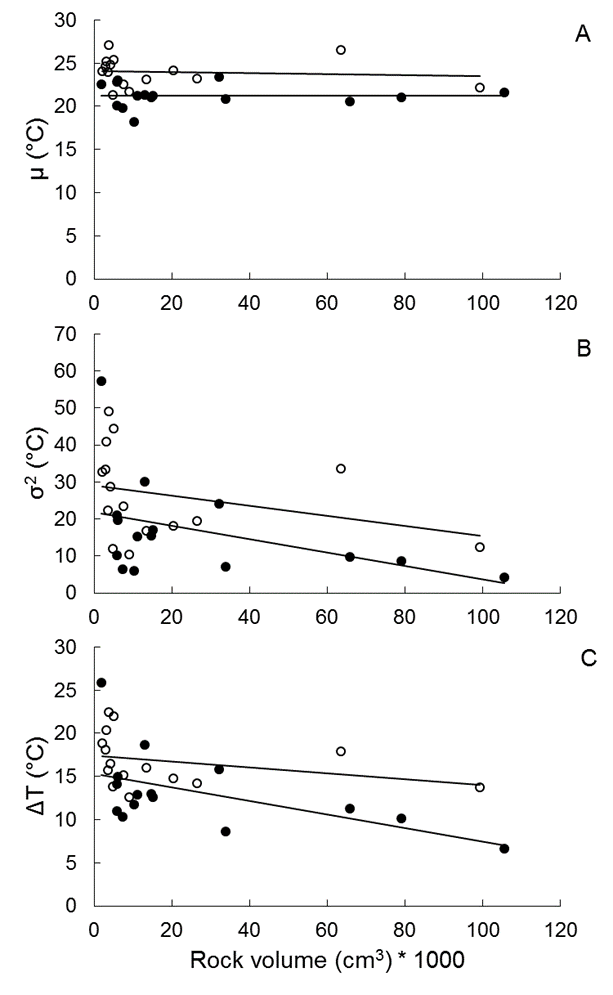

Supplement: S5 Fig — Chiricahua rocks are represented as open circles, while Pinaleño rocks are represented as closed circles. Note how thermal σ2 and ΔT decrease as rock size increases, while mean temperature remains relatively the same. However, R2 values are low (see Fig 2 for explanation): (A) Pinaleño R2 < 0.001, Chiricahua R2 = 0.011; (B) Pinaleño R2 = 0.179, Chiricahua R2 = 0.094; (C) Pinaleño R2 = 0.287, Chiricahua R2 = 0.097. (TIF) [file pone.0168105.s005.tif]

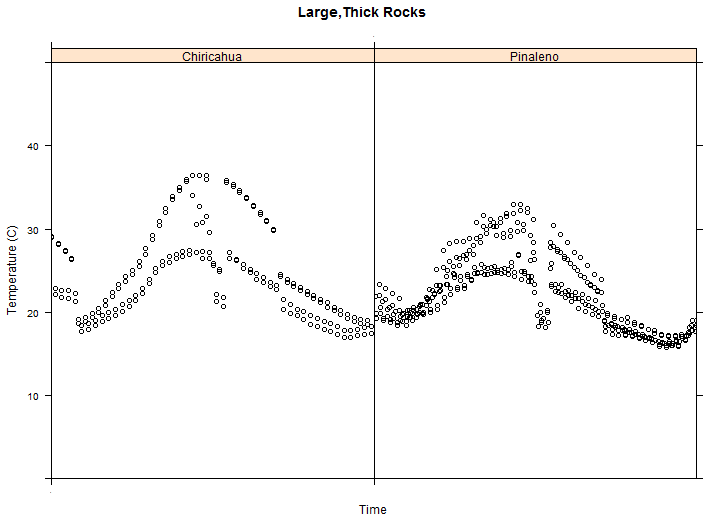

Supplement: S6 Fig — This graph represents temperature readings over a period of 48 hours. Although all sites were subject to the same rock size classification, site separation was necessary because of differences in elevation and canopy cover. Because the Pinaleño site had larger, thicker rocks than the Chiricahua site, more data was available, and the thermal profiles are substantially less variable than the Chiricahua thermal profiles. (TIFF) [file pone.0168105.s006.tiff]

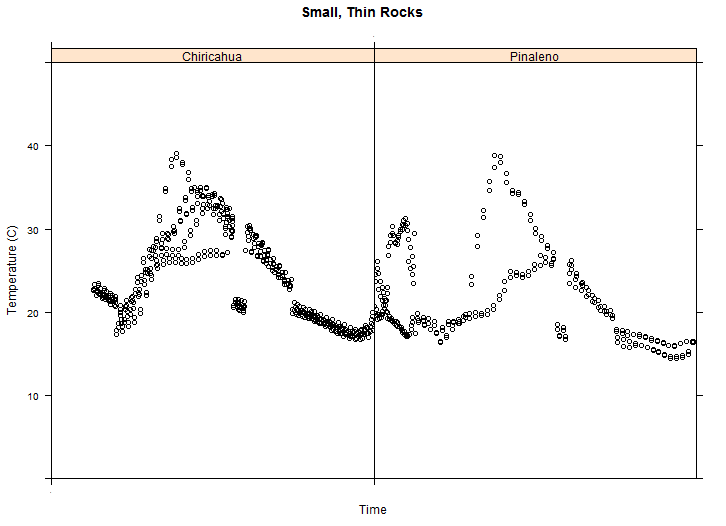

Supplement: S7 Fig — Like S6 Fig, this graph represents temperature readings over a period of 48 hours, and sites were separated because of differences in elevation and canopy cover. Because the Chiricahua site had a larger percentage of very small rocks, this site has more data available. However, note that very small, thin rock thermal profiles have significantly more variability than large, thick rocks. (TIFF) [file pone.0168105.s007.tiff]
